# Hepatitis B Vaccine Birthdose Practices in a Country Where Hepatitis B is Endemic — Laos, December 2011–February 2012

**Published:** 2013-07-26

**Authors:** Anonh Xeuatvongsa, Chansay Pathammavong, Kongxay Phounphenhack, Rita Reyburn, Keith Feldon, Melanie J. Thompson, Karen A. Hennessey, Minal K. Patel, Eyasu H. Teshale, Francisco Averhoff

**Affiliations:** Ministry of Health, Laos; World Health Organization, Laos; World Health Organization, Western Pacific Regional Office, Philippines; Global Immunization Div, Center for Global Health; Div of Viral Hepatitis, National Center for HIV/AIDS, Viral Hepatitis, STD, and TB

Chronic hepatitis B virus (HBV) infection causes approximately 325,000 deaths from cirrhosis and liver cancer each year in the Western Pacific Region of the World Health Organization (WHO) (1). With an estimated infection prevalence of >8%, HBV is considered highly endemic in Laos (2) and is most commonly transmitted from mother to child during birth and early childhood. A hepatitis B vaccine birth dose (HepB-BD) is needed to prevent mother-to-child HBV transmission (3). To assess gaps in coverage and identify possible remedies for improvement of coverage, during the 3-month period December 2011–February 2012, the Laos Ministry of Health and WHO staff members surveyed 37 health facilities in five provinces in Laos, inquiring about HepB-BD knowledge and practices among health-care providers and estimating HepB-BD coverage provided by the facilities. For facility-based births, the median HepB-BD coverage was 74% (interquartile range: 39%–97%). Hepatitis B vaccine was not in stock at 18 (49%) of the 37 facilities on the day they were visited. Of the 37 facilities, 17 (46%) assisted with home births, and 23 (62%) conducted postnatal home visits. Of the 17 facilities that assisted with home births, seven (41%) included HepB-BD vaccination as part of the service; of the 23 that conducted postnatal home visits, 15 (65%) provided HepB-BD as part of the visit. However, among those reporting that they provided these outreach services, only 48 births were recorded as attended, and only 81 postnatal visits were recorded as conducted during the 3-month period. Health facilities can help prevent mother-to-child HBV transmission in Laos by ensuring vaccine availability, vaccinating all infants born in the facility, and enhancing outreach services for home births.

Despite having only 28% of the world’s population, approximately half of all HBV-attributable deaths globally occur in the WHO Western Pacific Region (4). To control HBV transmission, the region adopted a goal of reducing chronic hepatitis B prevalence to <2% by 2012 among children aged ≥5 years and to <1% in a target year yet to be determined (5).

Administration of the hepatitis B vaccine birth dose followed by timely completion of the hepatitis B vaccine series is 70%–95% effective in preventing mother-to-child HBV transmission (6,7). During 2006–2011, reported HepB-BD coverage in Laos increased from 3% to 34% (8). Despite this increase, the country continues to have the lowest coverage in the region, largely because only 37% of women in Laos give birth with the assistance of a skilled birth attendant.*

This assessment was conducted in five of 24 provinces selected on the basis of accessibility and larger population size. In each of the five provinces, the central or provincial hospitals were selected, along with a sample of two district hospitals and four health centers to ensure representation of facilities with both high and low rates of HepB-BD coverage. One district health office that offered vaccination services also was included.

At each of the 37 health facilities, a standardized questionnaire was administered by in-person interview with one to three staff members, including administrators, staff members in charge of the delivery ward, and vaccination department personnel. Information collected by interview concerned staffing; job descriptions and training; policies and practices with regard to administering HepB-BD; administration of HepB-BD as part of outreach service delivery; and availability, supply, and storage of hepatitis B vaccine in the facility and for outreach delivery. Birth and vaccination registries were reviewed to obtain total birth and vaccination data for all births recorded by these facilities occurring during the 3-month period December 2011–February 2012. Data were extracted for newborns delivered in the 37 facilities and those receiving HepB-BD; among newborns receiving HepB-BD, information regarding birth setting (i.e., health facility or home) was obtained when available.

HepB-BD vaccination was defined as administration of the monovalent hepatitis B vaccine within the first 7 days of life, per the Laos vaccination schedule. For health-care facilities providing onsite delivery services, HepB-BD coverage was calculated by dividing the number of newborns who received HepB-BD in the facility by the total number of births in that facility. The median and interquartile ranges of facility-specific coverage rates also were calculated. A total of 37 facilities known to have reported HepB-BD vaccinations in 2011 were selected to visit during the study period (Figure). These facilities included seven central or provincial hospitals, nine district hospitals, one district health office, and 20 health centers.

## Facility-Based Births

Thirty-one (84%) of the 37 facilities reported providing birthing services onsite (Table). These 31 facilities had a total of 5,072 onsite births recorded during the 3-month assessment period, and all reported providing HepB-BD. At these facilities, 3,541 (70%) newborns received HepB-BD; median HepB-BD coverage was 74% (interquartile range: 39%–97%). HepB-BD was administered by skilled birth attendants at 19 (61%) of the 31 facilities. Four additional facilities without onsite birthing services reported providing newborns with HepB-BD, for a total of 35 (95%) facilities reporting provision of HepB-BD.

Vaccination practices varied (Table). Of the 35 sites administering HepB-BD, 10 (29%) relied on untrained staff members. Interviewees from 33 (89%) of 37 sites listed erroneous contraindications for hepatitis B vaccination at their facilities, including prematurity, low birth weight, jaundice, and having a mother with HIV infection (the only contraindication to hepatitis B vaccine is severe allergic reaction, such as anaphylaxis, following a previous dose or component of the vaccine). Of the 35 sites routinely administering HepB-BD, 13 (37%) reported not having vaccine in stock at some time during the 3-month study period, and 11 (32%) reported improperly discarding the second dose of the 2-dose HepB-BD vials. Eighteen (49%) of 37 facilities had no vaccine in stock on the day of the visit. Thirteen (42%) of 31 facilities providing delivery services did not provide vaccine at all times of day.

## Home-Based Births and Postnatal Visits

Of the 37 facilities visited, 17 (46%) reported assisting with home births, and 23 (62%) reported conducting postnatal home visits (Table). Seven (41%) of the 17 facilities providing home birth services also provide HepB-BD at the time of birth. Of the 23 facilities providing postnatal home visits, 15 (65%) provided HepB-BD as part of the visit. However, the facilities providing outreach services reported only 48 birth and 81 postnatal visits conducted during the 3-month period.

### Editorial Note

Median HepB-BD coverage among infants born in health facilities was only 74% in this assessment of 37 health facilities in Laos. This assessment identified low HepB-BD vaccination coverage rates and multiple challenges in HepB-BD implementation in health facilities in Laos, including vaccine stock outages, a lack of trained staff members to vaccinate newborns, and among staff members surveyed, common misperceptions about contraindications, all resulting in missed opportunities for vaccination. Additional challenges, given the large proportion of home births in Laos, include limited outreach birthing and postnatal services provided by the health facilities, which contribute to low HepB-BD coverage. These challenges also have been reported in other countries (9).

The results of this assessment highlight multiple opportunities for increasing HepB-BD coverage using existing health services: 1) focusing initial efforts on increasing coverage among newborns in health-care facilities, who are easier to access than those born at home; 2) ensuring vaccine stock availability so that HepB-BD prevention opportunities are not missed; and 3) integrating HepB-BD vaccination into ongoing home birthing and postnatal home visit services.

Vaccination coverage in Laos might be improved by designating a staff member responsible for implementing HepB-BD vaccination at each facility and ensuring that birthing professionals are provided with training. Such training could include emphasizing the importance of early vaccination in preventing mother-to-child HBV infection during birth (2). Training also could stress that there are no contraindications to HepB-BD except for known allergic reaction to the vaccine, which cannot be predicted at birth (3). All facilities should aim to administer HepB-BD within 24 hours of birth and, if this is not feasible, vaccine should be administered as soon as practical (2). Lack of vaccine onsite is another major barrier to vaccinating newborns in Laos. Understanding and correcting causes of vaccine stock outages are critical for increasing HepB-BD coverage.

Despite the small proportion of deliveries that occur in health facilities in Laos, implementing HepB-BD vaccination in health facilities is an efficient and practical approach to preventing perinatal HBV transmission (9). Making incentives to health-care workers contingent on provision of HepB-BD might be a feasible strategy to improve coverage in facilities (9). As more births occur in health-care settings, facility-based implementation of HepB-BD will become increasingly important.

Opportunities for increasing coverage to newborns born at home include integrating HepB-BD into existing maternal and newborn outreach activities. Policies supporting this activity are in place in Laos, including inclusion of HepB-BD vaccination in the recommended neonatal care package. However, outreach services remain limited because they are costly, underfunded, and highly dependent on external funding. As demonstrated in this assessment by the minimal number of home visits, outreach by a skilled provider is currently available to only a small proportion of new mothers. However, integrating HepB-BD vaccination with existing outreach activities is feasible by raising awareness, training staff members, ensuring the supply of vaccine, and taking advantage of the proven heat stability of hepatitis B vaccines, which allows for their use where refrigeration is not available (10).

What is already known on this topic?

Hepatitis B virus (HBV) infection is highly endemic in Laos, which has adopted vaccination strategies to reduce infection rates in children. A hepatitis B vaccine birth dose (HepB-BD), as part of a 3-dose schedule, is effective in preventing perinatal hepatitis B transmission. However, vaccination coverage with HepB-BD in Laos was estimated at only 34% in 2011.

What is added by this report?

In a survey of 37 health facilities conducted during December 2011–February 2012 in five of the 24 provinces in Laos, the median HepB-BD vaccination coverage among infants born in health-care facilities was 74%. Hepatitis B vaccine was out of stock at 49% of facilities at the time they were visited. Missed opportunities to vaccinate because of misunderstanding of vaccine contraindications and low rates of medical attendance at home births also were observed.

What are the implications for public health practice?

To reduce the prevalence of chronic hepatitis B infection in Laos, efforts could be directed at preventing missed opportunities for administration of HepB-BD by eliminating stock outages and dispelling misunderstandings about the vaccine among health facility staff members who attend to births. In addition, significantly improving overall birth dose coverage in the country will require ensuring inclusion of HepB-BD in home birthing and postnatal services, an increase in the proportion of home births that are medically attended, and an increase in postnatal home visits by medical staff members.

The findings in this report are subject to at least three limitations. First, the assessment covered only 37 health facilities in five of the 24 provinces in Laos and cannot represent the country as a whole. Second, facility-based birth dose coverage likely is overestimated because newborns born outside of the facilities who later received HepB-BD at the facilities could not be distinguished in most instances from those born in the facility who received HepB-BD. Finally, responses on the questionnaire could not be independently verified.

Laos has shown a strong commitment toward the Western Pacific Region goal of reducing chronic HBV infection prevalence in children aged ≥5 years to <1%. Such a reduction in HBV prevalence will require prevention of both perinatal and early childhood infections. Activities identified in this assessment can provide direction to help further strengthen efforts to prevent HBV infection in Laos.

## Figures and Tables

**FIGURE:**
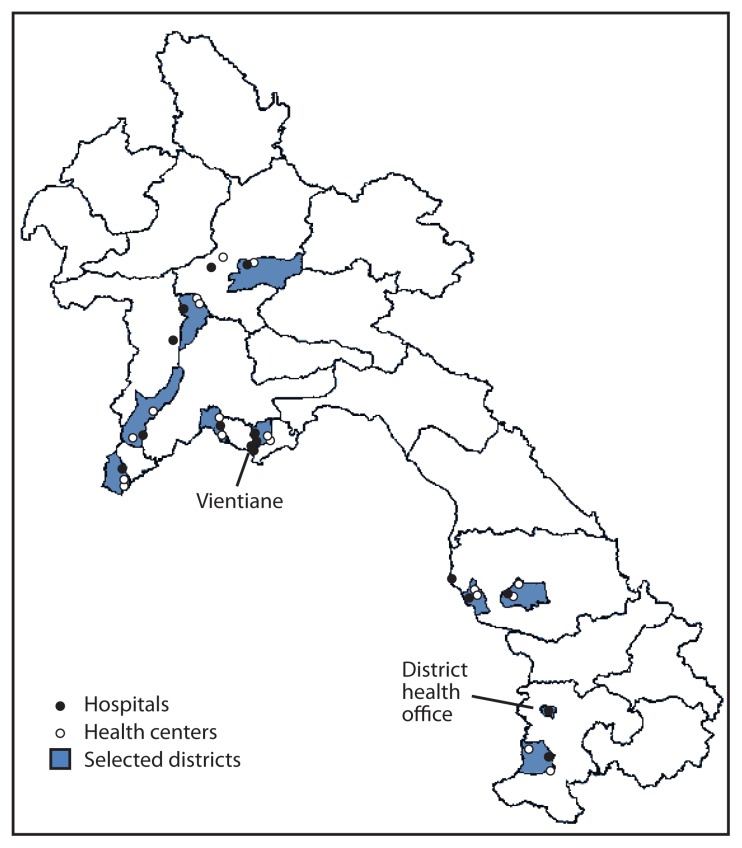
Location of selected districts and 37 health facilities surveyed regarding delivery services and hepatitis B vaccine birth dose (HepB-BD) practices — Laos, December 2011–February 2012 * Includes seven central or provincial hospitals, nine district hospitals, one district health office, and 20 health centers.

**TABLE:** Delivery services and hepatitis B vaccine birth dose (HepB-BD) practices at 37 health facilities* — Laos, December 2011–February 2012

Services/Practices	Total facilities asked	No.	(%)
**Birthing**			
Deliveries on site	37	31	(84)
SBAs not available at all times to deliver births	31	11	(35)
Length of stay after delivery >24 hrs	31	9	(29)
**Vaccination**			
Administers HepB-BD	37	35	(95)
Vaccination staff trained in HepB-BD	37	25	(68)
SBAs trained in HepB-BD	31	24	(77)
Report incorrect contraindications	37	33	(89)
Inappropriately discard second dose	35	11	(31)
Out of vaccine stock during assessment period	35	13	(37)
No stock on day of assessment	37	18	(49)
If facility delivers births, vaccine available 24 hrs/day	31	18	(58)
Administers HepB-BD in delivery room	31	16	(52)
**Home births and postnatal care**			
Staff members attend home births	37	17	(46)
Include HepB-BD vaccination at home births	17	7	(41)
Conduct postnatal home visits	37	23	(62)
Include HepB-BD at postnatal home visits	23	15	(65)

**Abbreviation:** SBA = skilled birth attendant.

* Includes seven central or provincial hospitals, nine district hospitals, one district health office, and 20 health centers.

## Footnotes

* Additional information available at http://www.moh.gov.la/index.php?option=com_phocadownload&view=category&id=9%3Aresearch&download=51%3Astatistic-him&Itemid=59&lang=en.
